# Facilitating Seed Iron Uptake through Amine-Epoxide Microgels: A Novel Approach to Enhance Cucumber (*Cucumis sativus*) Germination

**DOI:** 10.1021/acs.jafc.4c01522

**Published:** 2024-06-18

**Authors:** Felipe B. Alves, Heber E. Andrada, Bruno A. Fico, Julia S. Reinaldi, Denise C. Tavares, Iara S. Squarisi, Gabriel Sgarbiero Montanha, Laura G. Nuevo, Hudson W. P. de Carvalho, Carlos A. Pérez, Eduardo F. Molina

**Affiliations:** † Universidade de Franca, Av. Dr. Armando Salles Oliveira 201, Franca, SP 14404-600, Brazil; ‡ Grupo de Estudo em Fertilizantes Especiais e Nutrição, Centro de Energia Nuclear na Agricultura, Universidade de São Paulo, Av.Centerário 303, Piracicaba, SP 13400-970, Brazil; § Dipartimento di Biologia e Biotecnologie Charles Darwin, Sapienza Università degli Studi di Roma “La Sapienza”, Via dei Sardi 70, Roma 00185, Italy; ∥ Chair of Soil Science, Mohammed VI Polytechnic University, Lot 660, Ben Guerir 43150, Morocco; ⊥ Brazilian Synchrotron Light Laboratory, Brazilian Centre for Research in Energy and Materials, Rua Giuseppe Máximo Scolfaro, 10000, 13083-1000 Campinas, Brazil

**Keywords:** aliphatic polyether chains, sustainable agriculture, nutrient absorption, seed coat, synchrotron radiation-based X-ray fluorescence SRXRF

## Abstract

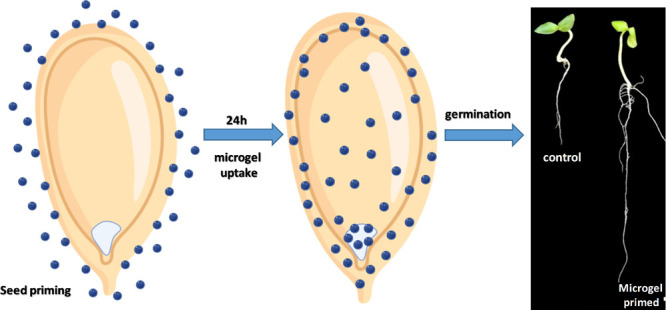

Enhancing the initial stages of plant growth by using polymeric gels for seed priming presents a significant challenge. This study aimed to investigate a microgel derived from polyetheramine-poly(propylene oxide) (PPO) and a bisepoxide (referred to as micro-PPO) as a promising alternative to optimize the seed germination process. The micro-PPO integrated with an iron micronutrient showed a positive impact on seed germination compared with control (Fe solutions) in which the root length yield improved up to 39%. Therefore, the element map by synchrotron-based X-ray fluorescence shows that the Fe intensities in the seed primers with the micro-PPO-Fe gel are about 3-fold higher than those in the control group, leading to a gradual distribution of Fe species through most internal embryo tissues. The use of micro-PPO for seed priming underscores their potential for industrial applications due to the nontoxicity results in zebrafish assays and environmentally friendly synthesis of the water-dispersible monomers employed.

## 1 Introduction

The continuous upscaling in the world population drives an increasing demand toward sustainable food production in an already strained environment.^1^ In this scenario, the development of novel engineered materials stands out as a crucial point toward more efficient technological applications in agriculture. Therefore, developing polymers may lead to new frontiers for more efficient operations in both the agricultural and industrial production of foodstuffs. The design of novel and multifunctional materials to improve the production quality and processing quantity of food products may help to close the gap between the two fields of polymeric materials and the areas of agriculture and food development.^2,3^

Polymeric systems are used in the most diverse forms in agriculture, especially in the controlled release of agrochemicals and as useful vehicles for plant protection and water uptake-inducing compounds.^4,5^ The use of polymeric materials in agriculture and horticulture has increased considerably, not only as a replacement for traditional materials but also as a significant improvement in technological processes in the growing of agricultural vegetables and crops and in agricultural equipment and drainage technology.^6−8^ The goal of using polymers to design novel technologies in agriculture and horticulture is concerned with growing more and better plants faster in less space and at lower cost.^9^

Recently, Sikder et al.^6^ brought attention to polymeric-based materials in their widely read article, emphasizing their relevance in precision agriculture applications. This comprehensive review outlined numerous potential opportunities, including the utilization of functional polymers as nanocarriers, controlled delivery of agrochemicals, development of hybrid biopolymers, and exploration of stimuli-responsive polymers, among other innovative avenues.

An interesting class of polymers is termed micro- and nanogels, characterized by an internal gel-like structure that swells when exposed to a dispersing solvent.^10−12^ The size of these microgel networks typically spans from several micrometers down to nanometers, often referred to as nanogels.^13,14^ These gels possess softness and responsiveness related to macromolecules, and they can crystallize based on volume fraction similar to colloids and exhibit the ability to adsorb to interfaces, thereby reducing interfacial tension, resembling surfactants.^15,16^ The properties of these materials can be finely adjusted by incorporating functional groups along their molecular backbone. This design aspect allows for the creation of polymeric nano- or microgels capable of producing smart materials with remarkable swelling capacity (water uptake) and sensitivity to factors such as pH, oxidation, and temperature. Due to these versatile characteristics, nano- and microgels have found extensive use in health-related applications, serving as integral components in biosensors and functioning as carriers for efficient drug delivery systems.^17−19^

In recent years, there has been significant focus on the utilization of micro and nanogels in agricultural contexts, aimed at enhancing both plant health and food production.^12^ Meure et al.^20^ introduced an innovative biohybrid microgel constructed from poly(allylamine), serving as an intelligent system designed to address chlorosis upon the foliar application of the microgel containing an iron (Fe) source. Their findings demonstrated a notable restoration of green pigment in iron-deficient cucumber plants, commonly referred to as “regreening”, achieved without inducing any adverse effects on the plants attributable to the microgels used. Dhiman et al.^21^ detailed the development of a chitosan-functionalized microgel characterized by a substantial insecticide loading capacity. Moreover, this microgel exhibits a noteworthy affinity for binding Fe^3+^ ions to leaves, presenting a sophisticated approach for the targeted delivery of Fe^3+^ ions essential for plant nutrition and care.

Amine-epoxide polymeric gels are derived in a catalyst-free aqueous environment from monomers, specifically bisepoxide and aliphatic polyetheramine. The use of these polymeric gels for agricultural purposes are novel and rare in the literature.^22^ The chemistry underlying the formation of amine-epoxide gels involves nucleophilic addition, where the amine component attacks the electrophilic carbon of the C–O bond, inducing its breakage and resulting in ring opening. This process relieves ring strain, typically yielding alcohol products. These hydrogel-like particles exhibit colloidal stability and demonstrate responsiveness to various biologically relevant stimuli, including oxidation, temperature, and pH variations.^23^

This study aims to explore and gain a comprehensive understanding of the functional capabilities of amine-epoxide microgels in agricultural applications. To the best of our knowledge, for the first time, we demonstrated the use of this class of amine-epoxide polymer networks applied as (i) material for seed germination (as priming agent) without phytotoxic effects, (ii) safety formulations for aquatic organisms by zebrafish toxicity assays, and (iii) a versatile diffusion-controlled Fe-based nutrient formulation for cucumber seed treatment. In this context, cucumber seeds were primed with unloaded microgels (as a negative control) to assess potential phytotoxic effects during germination tests. To assess the ability of these gels to act as carriers for a micronutrient specifically were investigated the permeability and diffusion of iron (Fe^3+^ ions) through the seed coat. To achieve this, concentrations of Fe (from 10 to 100 mg L^–1^) were incorporated into the amine-epoxide gels to observe their effect on the seed’s shoot and root development. The spatial distribution of iron (Fe) at a tissue-specific level within cucumber seeds was investigated using benchtop micro X-ray fluorescence (μ-XRF) and synchrotron radiation X-ray fluorescence (SRXRF) techniques. The seeds were previously sown with an amine-epoxide gel containing Fe^3+^, while an Fe^3+^ solution was used as a control. These methodologies facilitated spatially resolved metal analysis, enabling a more comprehensive understanding of how the loaded polymeric gels influence the accumulation and distribution profile of iron (Fe) within the tissues of cucumber plants

Here, the polymeric gels are synthesized through the reaction of a polyetheramine containing a poly(propylene oxide) (PPO) backbone with a diepoxy poly(ethylene glycol) (DPEG) in an aqueous medium. Hence, we assessed the influence of these gel particles on seed germination and plant growth. Accordingly, this study aimed to (i) synthesize amine-epoxide gels utilizing environmentally friendly solvent-water, (ii) characterize the size/surface charge of the polymeric gels using dynamic light scattering (DLS) and zeta potential analysis, as well as analyze their morphology via transmission electron microscopy (TEM), and (iii) evaluate the toxicity of the gels through zebrafish assays, employing adult *Danio rerio*.

## 2 Materials and Methods

### 2.1 Materials

Polyetheramine containing a propylene oxide backbone (Jeffamine T-403 PPO-based *M*_w_ = 440 g mol^–1^) was kindly provided by Huntsman Chemical. Diepoxy poly(ethylene glycol) (DPEG, C_3_H_5_O_2_-(C_2_H_4_O)_*n*_-C_3_H_5_O, average *M*_w_ = 500 g mol^–1^), and iron source based on Fe-sodium ethylenediaminetetraacetate (Fe-EDTA) were purchased from Sigma-Aldrich. All reagents were used as received. Ultrapure water with a resistivity of 18.2 MΩ·cm was used during the synthesis of polymer gel.

### 2.2 Preparation of the PPO–DPEG Microgel

**Scheme 1:**
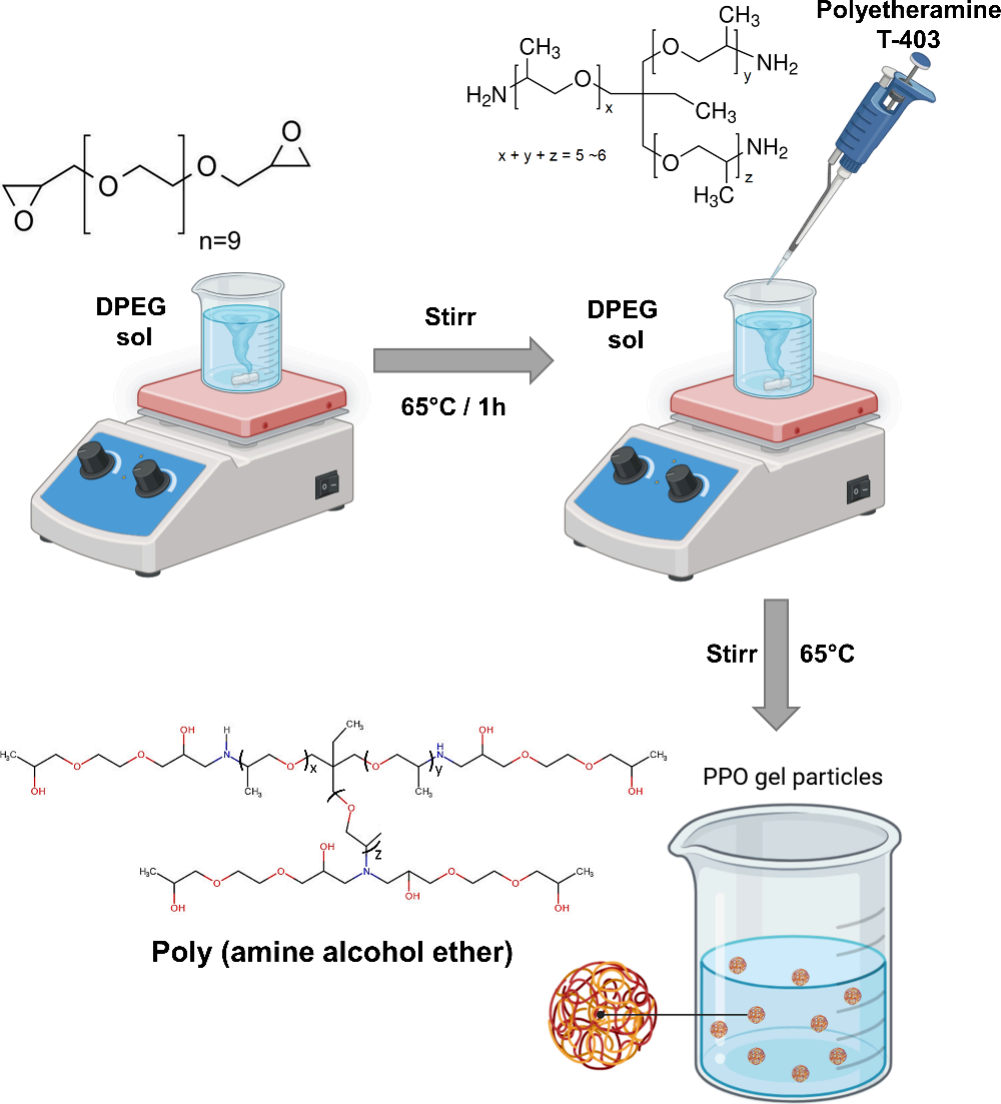
Synthetic Route for the Preparation of Micro-PPO Gel Based on DPEG and PPO T-403, where *x* + *y* + *z* = 5–6.

Poly(amine alcohol ether) microgel was synthesized by amine-epoxide “click” reaction^23−27^ by using water-soluble monomers PPO and DPEG. Briefly, DPEG (0.8 g) was dissolved in deionized water (10 mL) and left to stand for 60 min at 65 °C, under stirring. After, PPO-based polyetheramine (0.2 g) was slowly added to the DPEG solution and continued to react for 30 min. The resulting solution was cooled to room temperature and kept refrigerated at 2 °C. The monomer concentration in water was 10 wt % with a stoichiometric ratio PPO:DPEG of 1:3. The obtained microgels were named micro-PPOor micro-PPO-Fe (embedded with Fe-EDTA). Finally, the micro-PPO gel was purified by dialysis in water by using regenerated cellulose membranes. The embedded micro-PPO gel was formulated by dissolving a 10 mg portion of the lyophilized gel in 10 mL of the Fe solution (at the desired concentration). Iron(III)-sodium ethylenediaminetetraacetate is a commonly employed substance in agricultural applications. A schematic of the micro-PPO gel obtaining process is illustrated in Scheme 1.

### 2.3 Characterization of the Micro-PPO Gel

ZSU3100 Zetasizer Lab Blue (Malvern Instruments) equipped with an OBIS solid-state laser source (λ = 633 nm) was used to measure the hydrodynamic diameter (*D*_h_), polydispersity index (PdI), and zeta potential (ζ) of micro-PPO gel. The experiments were conducted at room temperature (∼25 °C) and repeated at least three times, and the dates were expressed as mean ± standard deviation (SD) (*n* ≥ 3). Transmission electron microscopy TEM (JEM 100CXII JEOL instrument) operating at 100 kV was used to assess the morphology of micro-PPO gels. For this, the aqueous polymeric gel was transferred to a copper grid.

### 2.4 Zebrafish Acute Toxicity Assessment

Adult zebrafish (*Danio rerio*; 6 months old, body weight 0,36 ± 0,09 g, body size 3,60 ± 0,26 cm) was acquired from a local commercial source and was maintained in stock aquaria with mineral water and aeration for 14 days before the assays. Fish maintenance conditions are described in Aldana-Mejía et al.^28^ The physicochemical characteristics of the water are shown in Table S1. The fish were exposed for 96 h in a static system, and the micro-PPO gel concentrations were 50, 75, and 100 mg L^–1^ with seven fish per group for each concentration. The negative control (only water) was included. Considering that OECD 203 does not establish a reference positive control, standardized propolis extract^29^ at a concentration of 25 mg L^–1^ was used as a reference control, which presents high toxicity in adult zebrafish.

Reference control (standardized propolis extract at a concentration of 25 mg L^–1^) groups were included. Considering that OECD 203 does not establish a reference positive control, standardized propolis extract^29^ was used as a reference control which presents toxicity. Thus, this was done to check the sensitivity and reproducibility of the batch experiments. During the treatment period with the micro-PPO, the animals were observed 24, 48, 72, and 96 h, after the beginning of the exposure, the mortalities and visible abnormalities were analyzed. All assays involving adult zebrafish followed the OECD 203 guidelines.^30^ The choice of micro-PPO concentrations for adult zebrafish assays followed the OECD guidelines,^30^ which suggests a limit of 100 mg L^–1^ of the substance. The experimental protocols used were submitted and approved by the Ethics Committee on the Use of Animals at the University of Franca (CEUA n° 2985080121). The experiments included males and females (*n* = 35). After the observations, the animals were euthanized with benzocaine (1:20,000) diluted in 98° ethyl alcohol (0.1 g mL^–1^). Also, the potential genotoxicity effect of micro-PPO was tested through the micronucleus test. The surviving fish population was used for the evaluation of genotoxicity by a micronucleus test (MN) in peripheral blood. The MN test was performed according to Baršiene et al.^31^ Briefly, a small drop of blood was collected by caudal puncture, which was immediately spread on clean glass slides, allowed to air-dry, fixed in absolute methanol for 20 min, and stained with 10% Giemsa for 10 min. Two slides were prepared per fish. The frequency of micronuclei (MNi) in erythrocytes was evaluated by scoring 5000 intact cells per fish at 1000× magnification. MNi was identified as structures with the following morphological characteristics: (1) spherical or ovoid extranuclear bodies in the cytoplasm, (2) a diameter of 1/3–1/20 of the main nucleus, (3) nonrefractory bodies, (4) similar color texture to that of the core, and (5) bodies completely separate from the main core.^32^ The frequency of micronuclei (% MNi) was calculated according to Nwani et al.^33^ calculated as follows:
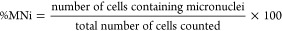


The results were analyzed using GraphPad Prism version 6, employing ANOVA and Tukey’s test with a 95% confidence level to detect significant differences between treatments. The results were expressed as the mean.

### 2.5 Seed Germination Assays

To examine the potential impact of the synthesized amine-epoxide on seed germination and seedling development, cucumber seeds were sown within the polymeric gel systems (experimental treatment) and pure water (control treatment). Cucumber seeds (*Cucumis sativus*) were subjected to surface sterilization through sequential 15 min washes in 2% sodium hypochlorite, followed by triple rinsing in deionized water. The experiment involved the application of the micro-PPO gel (pure or embedded with Fe ions) solution to observe the growth progress of cucumber plant shoots and root heights during seed germination. Specifically, 21 seeds were immersed in a flask containing 30 mL of the micro-PPO gel, allowing the flask to stand in darkness at room temperature for 24 h before germination on Petri dishes. Deionized water (excluding polymeric gel) was used as the negative control. The treated seeds (with either micro-PPO gel or water) were arranged on filter paper within Petri dishes, with three seeds per dish. Seven replicates were carried out for each treatment in the assays (total 21 seeds). The dishes were sealed to prevent water loss. The seed assay involving iron (Fe) followed the previously mentioned procedure. In this experiment, Fe-EDTA solution served as the control (concentrations from 10 to 100 mg L^–1^) while another batch utilized embedded micro-PPO gel containing the same Fe concentrations and was used for comparative analysis.

### 2.6 Measurement of Spatial Fe Distribution by Micro X-ray fluorescence (μ-XRF)

Microchemical assessment of the spatial distribution of Fe was carried out by using microprobe X-ray fluorescence spectroscopy (μ-XRF). In this regard, cucumber seeds treated with Fe solution and embedded micro-PPO-Fe gel at 100 mg L^–1^ were manually cross-sectioned, fixed into polypropylene thin films (VHG, FPPP25-R3), mounted on X-ray sample cups (Chemplex no.1530), and immediately loaded into a μ-XRF spectrometer (Orbis PC, Edax, USA), according to the procedure described elsewhere.^34−36^ The spatial distribution of Fe was evaluated through either 64-point line-scanning or 800-pixel matrix maps recorded across the embryo and cotyledonary tissues of cucumber seeds by employing a 30 μm polycapillary-focused X-ray beam operating at 45 kV and 500 μA sieved by a 250 μm thick Al primary filter. The dwell time was 15 s point^–1^ and 1 s pixel^–1^ for the line scans and maps, respectively, and the XRF spectra were recorded using a 30 mm^2^ silicon-drift detector (SDD) with a dead time of <2%. XRF line scans were conducted using three independent biological replicates, and the maps were recorded in one replicate. Deionized water was used as a negative control. Figure S1 details all of the procedures employed. Only XRF intensities above the instrumental limit of detection (LOD) were calculated as demonstrated by Montanha et al.^35^ were considered. The Fe intensities recorded across each seed tissue (seed coat, embryo, and cotyledon) were obtained from the line scans. The obtained data were compared through Kruskal–Wallis one-way analysis of variance followed by Dunn’s post hoc test at a 95% confidence level (*p* < 0.05). All analyses were conducted using the Prism software (version 9.2.0, GraphPad, USA).

### 2.7 Distribution of Fe in Seeds by Synchrotron Radiation X-ray Fluorescence (SRXRF) Analysis

To analyze the distribution of Fe across the seed coat and embryo interface, cucumber seeds treated either by Fe solution or micro-PPO gel embedded with Fe were prepared according to the procedure employed by Montanha et al.^37^ Briefly, the seeds were face scalped using a razor blade, embedded in optimal cutting temperature (OCT) medium (Tissue Plus, Fischer HealthCare, USA), and immediately plunged into liquid isopentane (Dinâmica, Brazil) supercooled with liquid N_2_. The cryofixed blocks were fixed on an adhesive cellophane film (Fitar, Brazil) and cut using a cryostat (CM1850, Leica, Wetzlar, Germany) at −25 °C (Figure S2), and the resulting 30 μm thick cross-sections were immediately placed in the XRF sample holder sealed with a 6-μm thick polypropylene (FPP25-R3, VHG, USA) thin film (Figure S3a). The samples were kept at −20 °C until the measurements, and the integrity of the cross-sectioned materials was assessed before and after the XRF measurements using a stereo microscope (SZX7, Olympus, Japan).

XRF analyses of the sampled seed cross sections were performed at the Tarumã endstation of the Carnaba coherent X-ray nanoprobe beamline of the Brazilian Synchrotron Light Laboratory (proposal ID 20231393). The beamline features a horizontal deflection four-bounce crystal monochromator, two four-element silicon drift detectors (Vortex-ME4, Hitachi High-Technologies Science America, USA), and a Kirkpatrick–Baez (KB) achromatic optic providing a 150 nm-wide X-ray focused beam spot. The XRF maps were recorded in the flyscan mode at a Zn K-line excitation energy (9750 eV) through 240 × 640 μm panoramic images. The measurements were performed at room temperature as no evidence of sample dehydration or structural collapse was observed within the measurement time frame (Figure S3b,c). The data were processed using the PyMCA software (version 5.6.3, ESRF Software Group, France).^38^ The elemental intensities found at each pixel unit were normalized by the ring current (*I*_0_) and fitted according to the beamline instrumental parameters. The obtained outputs are presented in Figure S4 and Table S2. Two independent biological replicates were used for each treatment, except the positive control (Fe solution), as detailed in Figure S5. The XRF images and profile of Fe intensities recorded across the seed coat and embryo tissues were obtained using Fiji^39^ and Prism software (version 9.4.0, GraphPad, USA).

## 3 Results and Discussion

### 3.1 Characterization of Micro-PPO Gel

**Figure 1:**
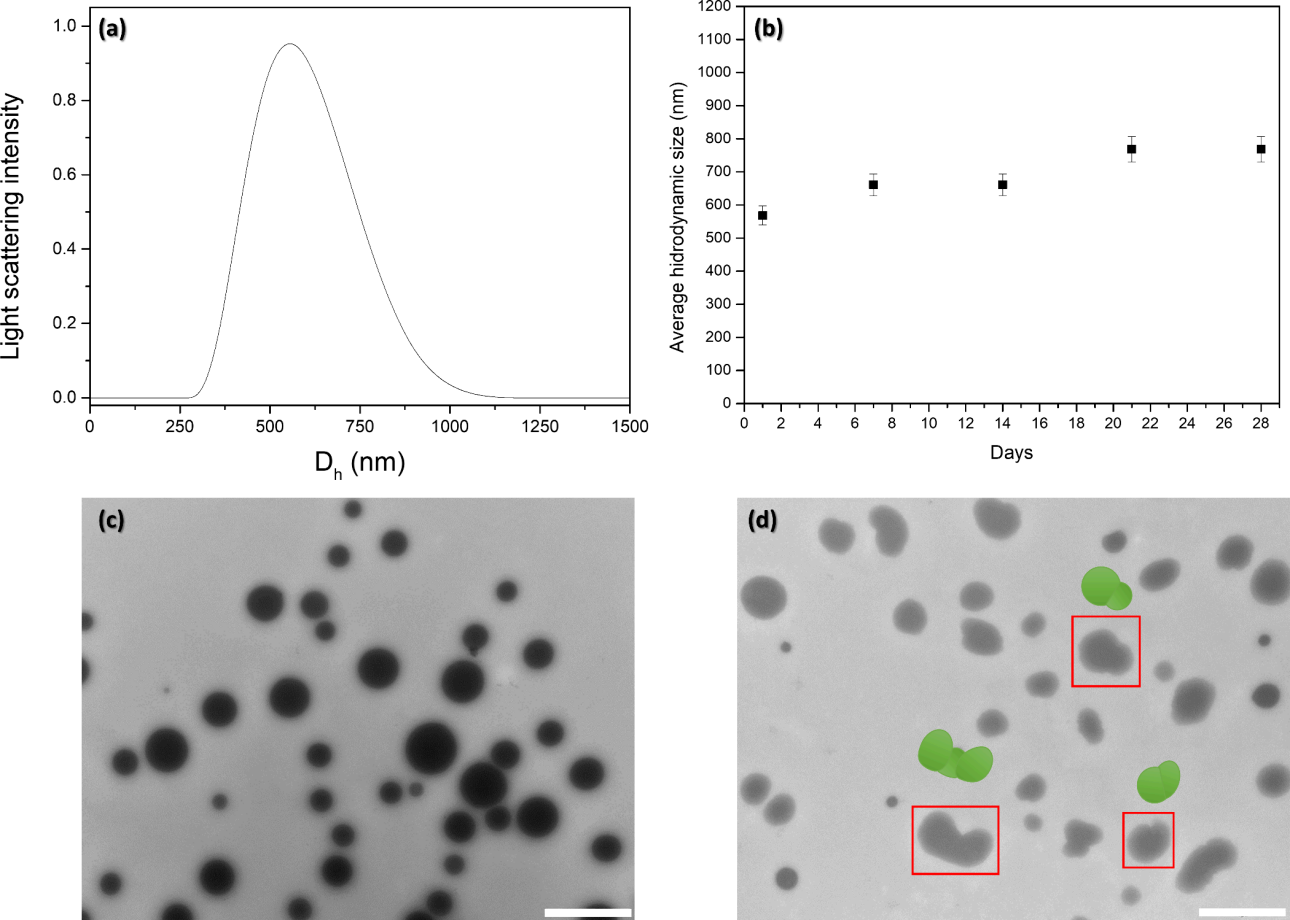
(a) Size distribution (hydrodynamic diameter, *D*_h_) of the micro-PPO after 1 day of synthesis; (b) stability of micro-PPO as a function of time. The change in the *D*_h_ (standard deviation) of three different samples as a function of time is shown; (c, d) Representative of TEM images of micro-PPO gel after 1 and 28 days, respectively. The scale bar observed in the images corresponds to 1 μm.

Dynamic light scattering (DLS) and transmission electron microscopy (TEM) were used to characterize the size, morphology, and stability of the polymeric micro-PPO gel. As shown in Figure 1a, the micro-PPO gel obtained had a *D*_h_ of 560 (±30 nm). The polydispersity of the particles (PdI) and the zeta potential (ζ) obtained were 0.30 and +14 mV (positively charged), respectively. It was observed good stability of the nanogels (during the initial 14 days) ranging from 560 to 650 nm, which showed an increase in *D*_h_ (reaching approximately 770 nm) after 21 and 28 days, respectively (see Figure 1b). The TEM images of micro-PPO are shown in Figure 1 (low panel), which illustrates the surface morphology of the microgels after 1 day (Figure 1c) and 28 days (Figure 1d) of synthesis, respectively. The microgels showed well-defined spherical morphology having particles with sizes of 520 (±20 nm) and 380 (±20 nm) (Figure 1c). Within 28 days after synthesis, dumbbells and snowman particles can be observed, leading to particle aggregation. The TEM results stand following the DLS measurements.

### 3.2 Micro-PPO Gel Toxicity

**Figure 2:**
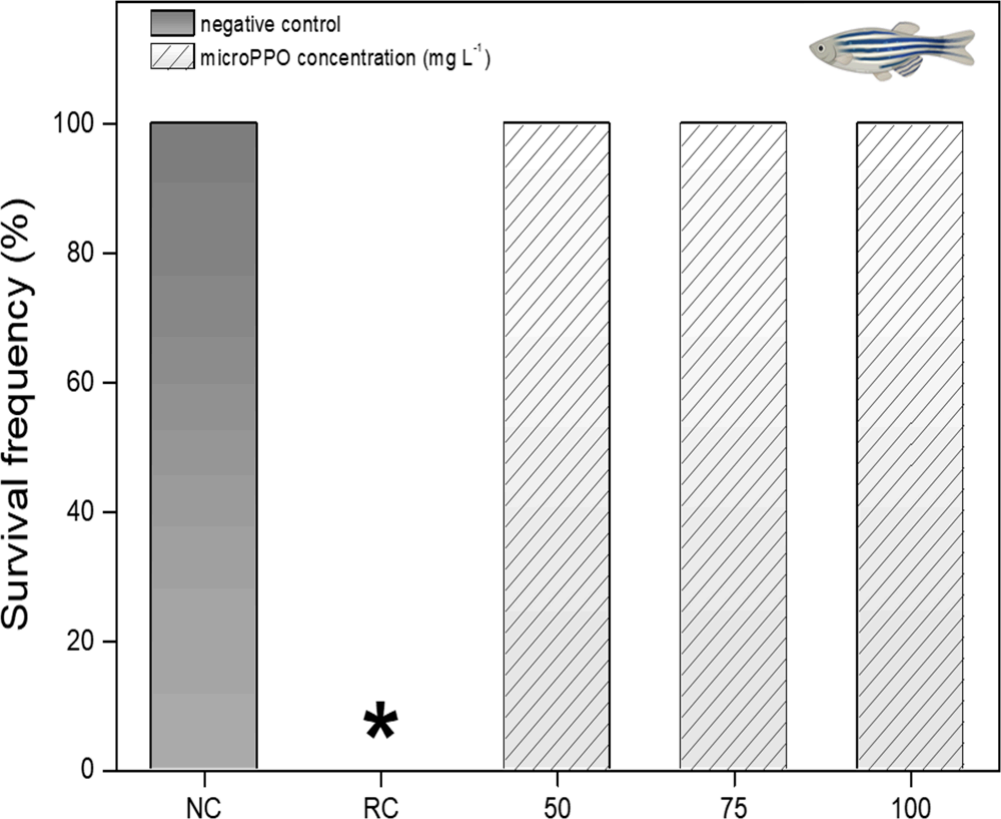
Survival frequency of zebrafish exposed to different concentrations of micro-PPO (50; 75 and 100 mg L^–1^) and their respective controls after 96 h. NC, negative control (water); RC, reference control (standardized red propolis extract −25 mg L^–1^). *Significantly different from the negative control group (*p* < 0.05).

We evaluated the toxicity of micro-PPO in adult zebrafish, as shown in Figure 2. As a direct exposure to microgel formulation (concentrations from a low 50 mg L^–1^ to a higher 100 mg L^–1^), no significant aquatic toxicity (without fish mortality) was observed after 96 h, which suggests that the obtained micro-PPO gel has high safety for aquatic organisms. This characteristic of the obtained gel opens positive perspectives for poly(amine alcohol ether) as carriers in agricultural applications such as seed priming and foliar treatment.

For the genotoxicity test (nuclear abnormalities in erythrocytes), the micro-PPO gel at concentrations (50, 75, and 100 mg L^–1^) demonstrated no genotoxic effects in peripheral blood of zebrafish (Table 1). The frequency of erythrocytic micronuclei in the fish exposed to the micro-PPO gel showed no statistically significant effect compared to the control at 4 days (96 h) of exposure. Nuclear abnormalities observed in erythrocytes of zebrafish (Figure S6) were only micronuclei, which for the total nuclear abnormalities (frequency), no significant micro-PPO exposure effects (*P* > 0.05) were found compared to the control (water) at 4 days of exposure. The literature has demonstrated that the exposition of structured materials could induce genotoxicity effects (nuclear abnormalities) in fish as the formation of micronuclei, nuclear buds, blebbed nuclei, and notched nuclei.^40^ For the first time, we showed the genotoxicity effect of amine-epoxide gels after exposure to adult zebrafish (*Danio rerio*), where these results support the use of micro-PPO for agricultural purposes.

**Table 1:** MN frequencies in Peripheral Blood of Zebrafish Exposed to Micro-PPO with Concentrations from 50 to 100 mg L^–1^ and Respective Negative Control (Water)

	**% MN frequencies**	
**treatment**	concentration (mg L^–1^)	96 h
negative control^a^		0.013
micro-PPO^a^	50	0.013
micro-PPO^a^	75	0.006
micro-PPO^a^	100	0.026

a A total of 15.000 cells were analyzed per treatment group.

### 3.3 Effects of Micro-PPO Gel on Cucumber Seed Germination

Seed priming has been widely employed to enhance seed germination rate and plant development.^41^ Cucumber (*Cucumis sativus*) holds a prominent position among the globally cultivated and consumed vegetable crops, commonly used as a model for agricultural studies, and ranking among the top four most extensively cultivated vegetables worldwide, following tomatoes, cabbages, and onions.^42^ In this regard, cucumber seeds were primed (for 24 h) with micro-PPO to evaluate the effects of the gel on seed germination potential as well as root and shoot length over time. For comparison, deionized water served as the control treatment (hydropriming). Following seed priming (using micro-PPO or water), the shoot and root development (length) were monitored for 12 days. The results showed that micro-PPO induced the germination of cucumber seeds following the control (DI water) behavior (Figure S7). This observation, along with nontoxicity to the animals (zebrafish assays), indicates that the micro-PPO has the potential to be applied in agriculture without negatively impacting plant growth. Zhou et al.^43^ applied nanochitin (NC) to tobacco to evaluate the bioactivity and potential of the NC suspensions on seed germination. The authors demonstrate that NC exhibits a robust capacity to enhance seed germination and promote plant growth by using low suspension concentrations (about 0.004% w/v). Zhang et al.^44^ demonstrated the preparation of cellulose anionic hydrogels, serving as natural stimulants for seed germination and seedling growth. Giving the high hydrophilicity and hence water absorption capacity, polymer-based hydrogels exhibit a potential application for nutrient delivery to plants.

**Figure 3:**
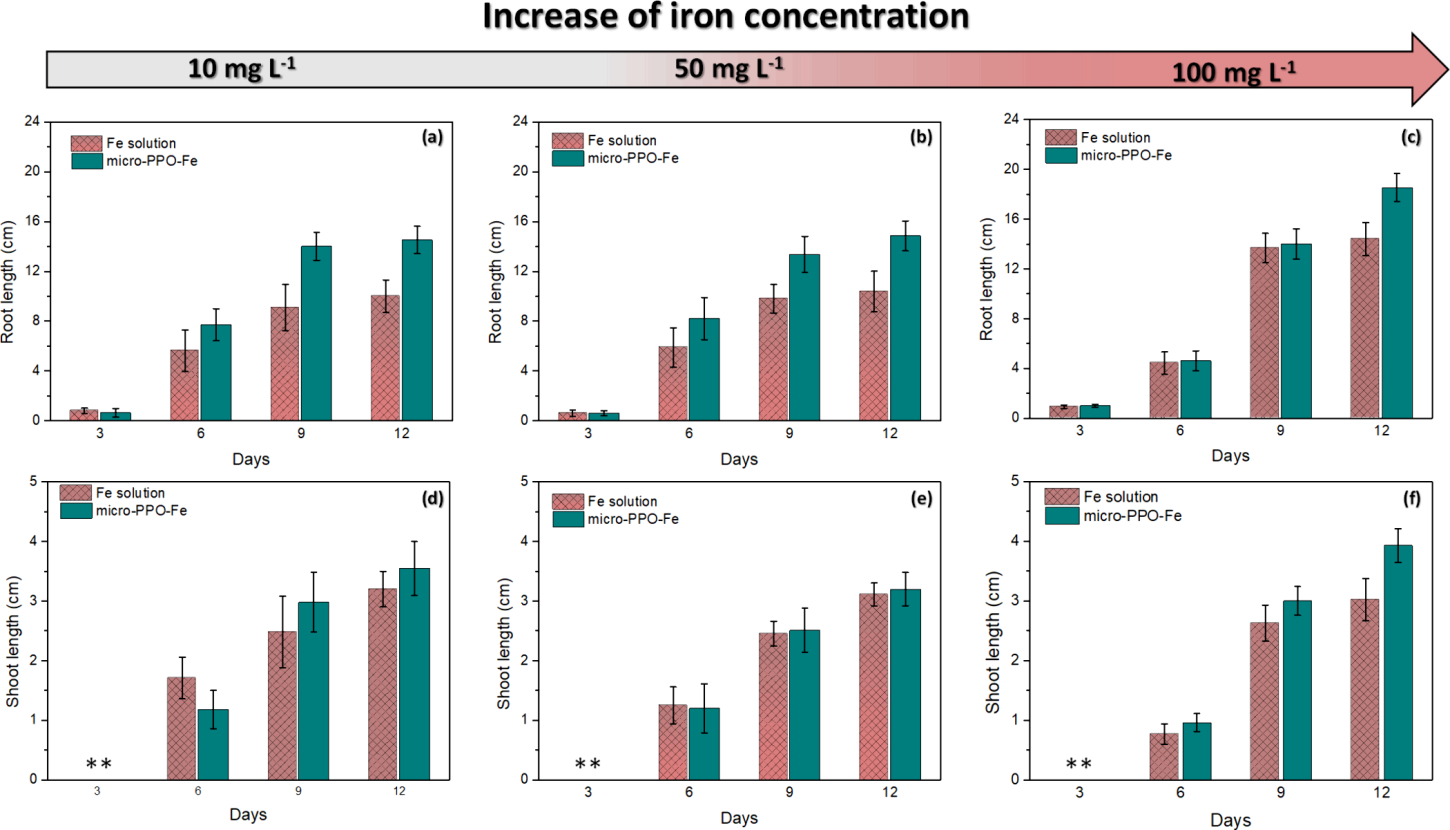
Influence of iron concentration on initial seedling growth during 12 days after sowing in Fe solution (controls) and micro-PPO-Fe gels. Development of (a–c) the root length and (d–f) shoot length as a function of iron concentration from 10 to 100 mg L^–1^. **Initial germination process with radicals emerging from the seeds. All of the results represent the average of seven Petri plates, containing 3 seeds per plate.

The results for the germination of seeds (root and shoot development) treated with Fe solutions and embedded micro-PPO-Fe are presented in Figure 3. Regardless of the iron concentration, a positive impact on seed germination was observed by micro-PPO-Fe treatments. Compared with control (Fe solutions), the root length of cucumber seeds was increased as a function of germination time by using micro-PPO-Fe gels (Figure 3a–c). We found root yield improvements of about 23% (Figure 3a), 39% (Figure 3b), and 32% (Figure 3c) on the 12th day (using micro-PPO-Fe as seed priming) containing 10, 50, and 100 mg L^–1^ of iron source, respectively. However, there was no significant difference in seed shoot length in comparison between the Fe solution (control) and micro-PPO-Fe (Figure 3d–f). In the case of shoot growth, only after the 12th day of treatment using 100 mg L^–1^ of Fe (see Figure 3f), there was an increase in the shoot size (∼29%) when comparing treatments using micro-PPO and Fe solution.

**Figure 4:**
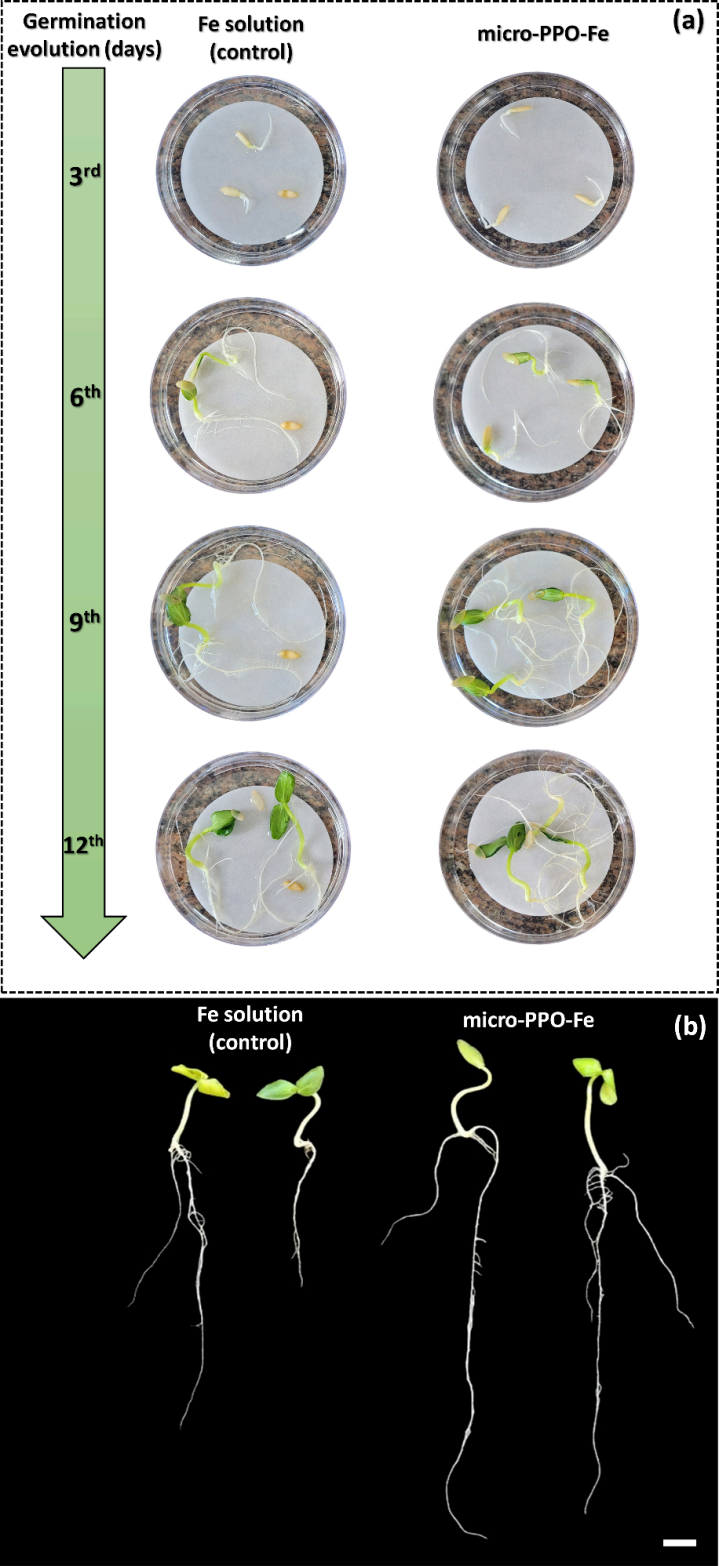
(a) Comparison of germination and development of cucumber seeds exposed to (left images) Fe solution as control and (right images) polymeric embedded micro-PPO-Fe gel during 12 days; (b) image of seedlings after 12 days. The Fe concentration used for both seed treatments (control and micro-PPO-Fe) was 100 mg L^–1^. Scale: 2 cm.

The enhancement of seed germination by embedded micro-PPO-Fe may be related to the diffusion of the gel particles (polymeric microgels) through the whole seed, with relatively high levels of Fe distributed into the compartments (e.g., coat, embryo, and cotyledon) of the seed. Likewise, micro-PPO gel could facilitate the uptake and absorption of nutrients by seed, leading to a synergic effect on the germination process. It can be noted that the increase of iron amount embedded into micro-PPO-Fe gel reflects an augment of germination potential over 12 days (see Figures 3 and 4, the development of germination of the cucumber seeds treated with Fe solution and micro-PPO-Fe both containing 100 mg L^–1^ of nutrient). Thus, to confirm our hypothesis, seeds treated with micro-PPO-Fe gel containing 100 mg L^–1^ (see results from Figure 3c,f) were chosen and in-depth studied by X-ray fluorescence (μ-XRF and SRXRF) techniques.

### 3.4 Spatial Distribution of Fe in Cucumber Seeds by Micro-PPO Gels: μ-XRF and SRXRF Studies

From a biological standpoint, seeds are crucial to the renewal of the species and thereby exhibit a conserved structure that serves as protection to ensure its maximum germination potential.^35^ Hence, to assess whether the effects observed on germination are related to seed Fe-based priming, both benchtop and synchrotron-based micro and nanoprobe X-ray fluorescence spectroscopy (XRF) were employed to assess the Fe localization throughout the cucumber seed tissues. XRF is a powerful analytical tool that has been widely explored in a myriad of seeds, such as maize,^37,44^ soybeans,^36,45,46^ kidney beans,^35,47^ cowpea,^48,49^ and tomato.^48^

**Figure 5:**
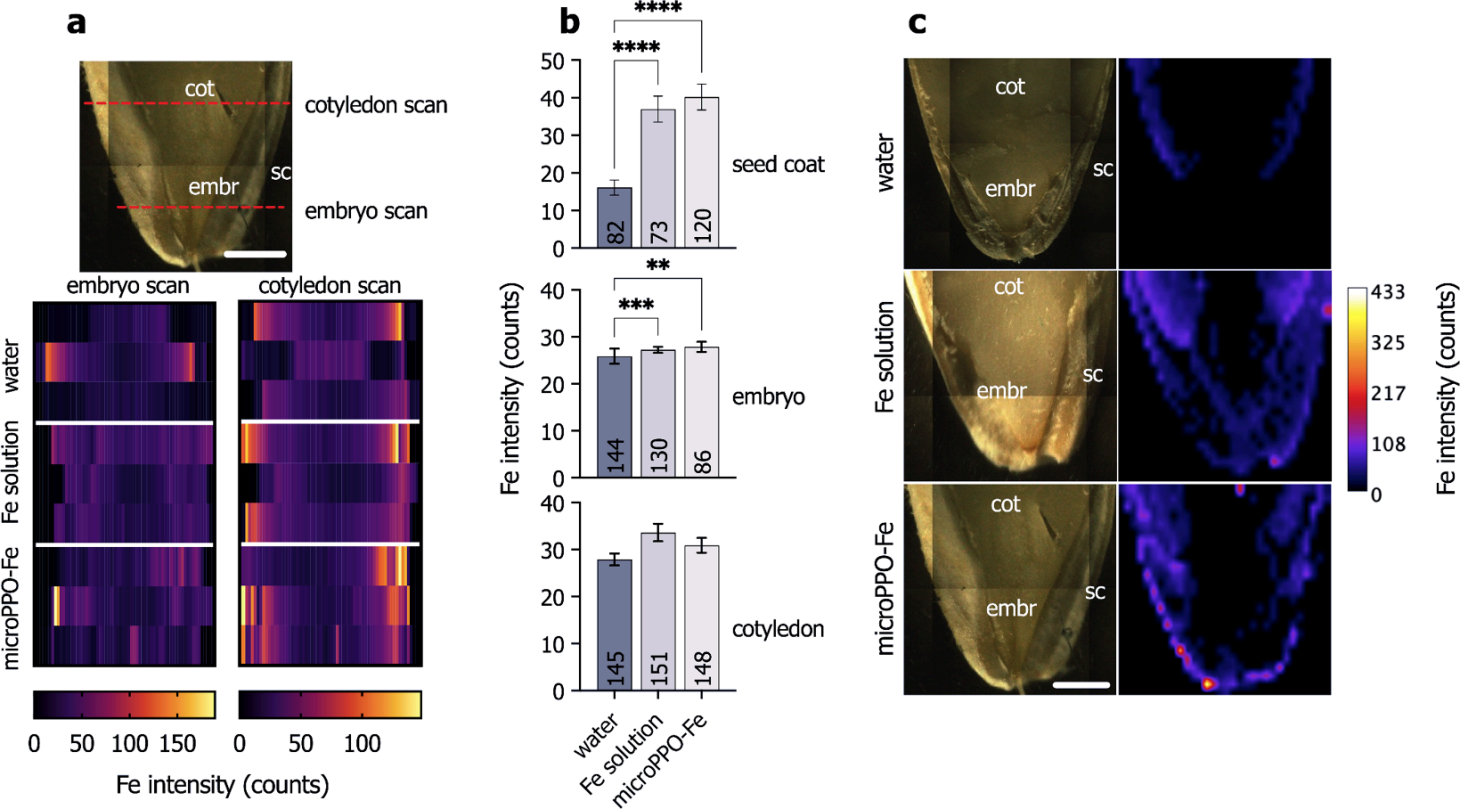
Microprobe μ-XRF scanning of Fe distribution in cucumber seed cross sections primed with either the negative (water) and positive controls (Fe solution) or microPPO-Fe gel solutions. (a) The scanned areas are indicated by the red dashed line in the schematic photograph. (b) The resulting Fe intensities recorded in all observed seed tissues, that is, seed coat (sc), embryo (embr), and cotyledon (cot), were compared across the treatments. The bar indicates the mean and standard error of several measurements recorded in three independent biological replicates. The *n* is indicated at the bottom of each bar. The data were subjected to Kruskal–Wallis one-way analysis of variance followed by Dunn’s post hoc test at a 95% confidence level (*p* < 0.05). (c) The embryo of cucumber seeds exhibited higher Fe content, as confirmed by the XRF maps showing the spatial distribution of Fe throughout the seed tissues. Scale: 1 mm.

Figure 5 shows the spatial distribution of iron in cucumber seeds cross sections obtained by benchtop XRF. The line scans (see Figure 5a) reveal that Fe intensities increase from the seed coat toward cotyledon and, most notably, the embryo tissues. Additionally, there was a consistent increase in Fe counts in both the positive control (Fe solution) and the microPPO-Fe gel. This is confirmed by the comparative analysis of the Fe signals obtained in the seed coat, embryo, and cotyledon tissues (see Figure 5b), as well as the XRF mappings (see Figure 5c). It shows that both the seed coat and embryo from the seeds primed with the embedded micro-PPO-Fe gel exhibited a higher Fe intensity compared to the negative control (water), whereas it was virtually the same in the cotyledons.

**Figure 6:**
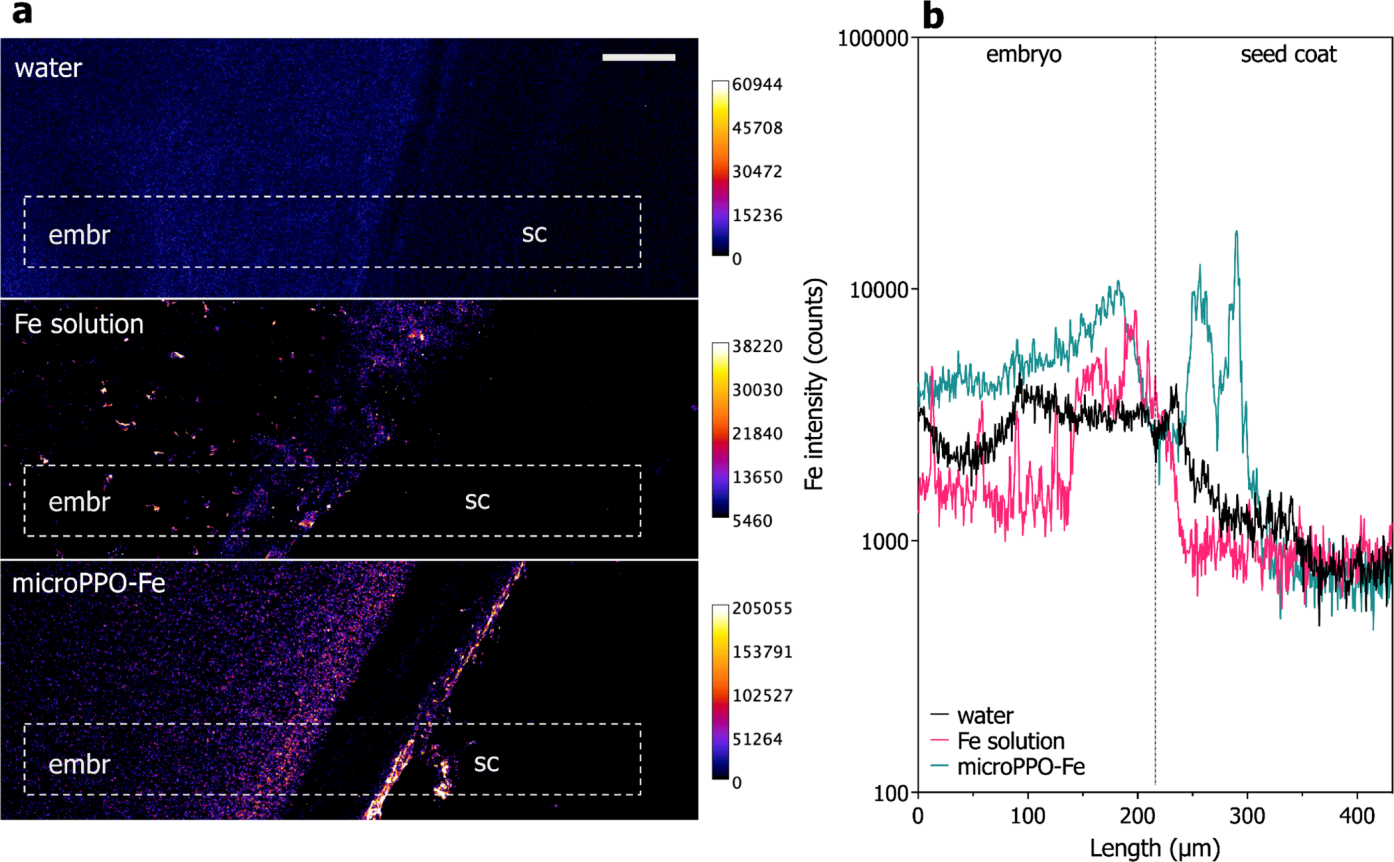
Synchrotron-based XRF maps of Fe distribution in cryofixed cross sections of cucumber seeds primed with either the negative (water) and positive controls (Fe solution) or the microPPO-Fe gel solutions (a). Fe profile across the seed coat (sc) and embryo (embr) interface within the dashed box (b). Scale: 50 μm.

Nevertheless, it is important to highlight that the lateral resolution provided by the 30 μm polycapillary-focused X-ray beam spot makes it difficult to properly compare the Fe intensities at interface regions. In this scenario, synchrotron radiation stands out as one of the foremost sources renowned for its ability to attain the best geometric resolution and detection limits. The excitation mode is exceptionally well-suited for examining biological samples within the micrometer to the millimeter size range.^50^ Therefore, synchrotron-based XRF was employed to precisely determine the spatial distribution of Fe at the seed coat–embryo interface. A comparison of the seed primers with water, Fe solution, and micro-PPO-Fe (at the concentration of Fe 100 mg L^–1^) is shown in Figure 6. The element map shows that the Fe intensities in the seed primers with the micro-PPO-Fe gel are about 3-fold higher than those in the negative control group (see Figure 6a) and depicts a clear Fe intensity gradient, detailed by the scans within the dashed boxes (see Figure 6b). This later revealed that seeds primed with the Fe solution and the micro-PPO-Fe gel exhibited maximum intensities at the seed coat, followed by a gradual decrease toward most internal embryo tissues. Figure S8 shows a similar trend observed on independent biological replicates.

Cucumber seeds exhibit a lipid and callose-coated single endosperm cell layer surrounding the seed coat (perisperm–endosperm envelope) that acts as a semipermeable barrier, limiting the transport of solutes toward the seeds.^51^ Our results suggest that the polymeric gels based on amine-epoxide, such as micro-PPO, may play a significant role in the permeability (diffusion dynamics of iron) through the perisperm–endosperm envelope to boost the germination process.

The SRXRF results indicate that a significant fraction of the Fe remains in the seed coat, that is, a lignin-based tissue that shields the embryo and cotyledon, against (a)biotic stresses, as previously observed in seeds exposed to micronutrients, such as Fe, Cu, and Zn.^45,47,52,53^ On the other hand, a higher Fe amount was also found in the embryo of the seeds explored to micro-PPO-Fe gel. Iron (Fe) plays a crucial role in various physiological processes of plants, such as photosynthesis and oxygen transport.^54,55^ Therefore, it is closely associated with the proper development of plants. Therefore, it is clear that the higher seed Fe uptake efficiency exhibited by the micro-PPO-Fe gel leads to improved germination and early seedling development of primed cucumber seeds.

The analysis of the Fe distribution maps obtained from synchrotron-based XRF (Figure 6) and the germination results (Figure 3) allowed for the proposed mechanism for the different behaviors observed after seed treatment (priming with Fe control and micro-PPO). A comprehensive anatomical characterization of cucumber seeds revealed that the radicle tip region’s perisperm–endosperm envelope presents less callose and, thereby, a higher permeability than the cotyledonary ones.^51^ It thereby indicates that structural differences are associated with the higher Fe absorption in the embryo tissues compared to the cotyledons in the seeds primed with the Fe control and micro-PPO solutions (Figure 5).

**Scheme 2:**
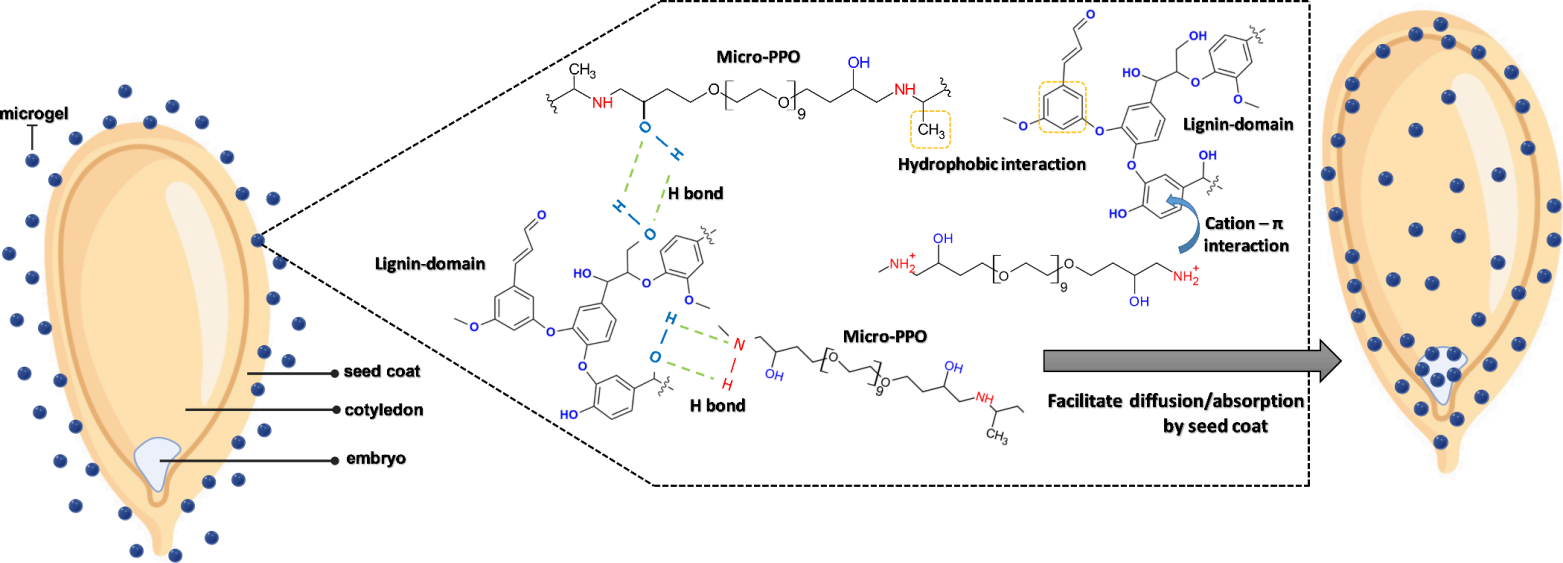
Mechanism of Absorption/Diffusion of Micro-PPO through Seed Coating Showing Possible Interactions (H Bond, Cation-π, and Hydrophobic Interactions) between Lignin-Rich Phase and Polymeric Gel

Furthermore, as cited above, lignin present in seed coat cells is hydrophobic and acts as a natural barrier that aids the cells’ osmotic equilibrium and membrane structure maintenance.^56^ It is important to note that after seed priming with micro-PPO gel, a large deposition of Fe ions in the seed coat followed by a more homogeneous distribution in embryo was observed (Figure 6a, lower panel). Since the polymer gel consists of poly(propylene oxide) (PPO), amine, and glycol groups, possible interactions between micro-PPO functional groups and lignin-rich phase influence a higher absorption of the solute (polymer gel) near the surface of the seed coat (see Scheme 2). Due to these possible interactions (H bond, cation-π, and hydrophobic interactions), the high amount of micro-PPO gel absorbed by seed coat can trigger two main effects: (i) establish an osmotic gradient that allows water to flow into the seed compartments and, as a consequence, (ii) a homogeneous deposition (or release) of Fe ions into the seed’s embryo. This effect is not observed when using Fe water solution control as seed priming (see Figure 6a, middle panel).

The mechanism proposed here agrees with the SRXRF data and supports the hypothesis that the utilization of microgels as a priming agent enhances seed germination. Notably, our research provides initial evidence of increased Fe distribution within the compartments of cucumber seeds through the application of an embedded polymeric micro-PPO gel. Since this is the first work that evaluate amine-epoxide gel as seed priming agent, the limitation of loaded Fe concentrations into micro-PPO (from 10 to 100 mg L^–1^) was based in the literature that demonstrated, for example, best root and shoot length results when used lower concentration of iron nanoparticles^57^ (∼5 mg L^–1^), and negative effects in germination when seeds (*Phaseolus vulgaris* L.) were exposed to Fe doses above 100 mg L^–1^.^58^

In conclusion, this research significantly advances our comprehension of polymeric microgels’ role as carriers that enhance the permeability of iron species within the compartments of cucumber seeds. Additionally, the methodologies employing benchtop μ-XRF and SRXRF illustrate their potential for evaluating the effects of polymeric gels on various farm seeds. Nontoxicity results observed in zebrafish and beneficial (priming) effects on cucumber seed by micro-PPO indicate that this class of polymer gel is safe for agriculture purposes. Furthermore, a preliminary test showed no phytotoxicity when the polymeric gel was sprayed onto cucumber leaves. It is clear that aspects encompassing the effectiveness of the present amine-epoxide gel on plant life cycle, as carrier of other mineral nutrients, and their effects on crop yield compared with conventional fertilizer sources need to be investigated in future. This forward-looking approach will deepen our understanding of the practical implications and potential applications of these microgels in agriculture.

## Supporting Information Available

Details of the samples and experimental setup employed for μ-XRF and SRXRF, iron (Kα and Kβ) lines fitting obtained on the synchrotron-based XRF mapping, and physicochemical properties of the test water for zebrafish assays and erythrocytes of zebrafish (PDF)
