# Supplementary material for: Facilitating Seed Iron Uptake through Amine-Epoxide Microgels: A Novel Approach to Enhance Cucumber (Cucumis sativus) Germination
Source: J Agric Food Chem. 2024 Jun 18;72(26):14570–80. doi: 10.1021/acs.jafc.4c01522 (PMC11229000; doi:10.1021/acs.jafc.4c01522)
Supplement: Supplementary file 1 — jf4c01522_si_001.pdf [file jf4c01522_si_001.pdf]

ELECTRONIC SUPPLEMENTARY MATERIAL

Facilitating Seed Iron Uptake through Amine-Epoxide Microgels: A Novel Approach to Enhance Cucumber (Cucumis sativus) Germination

Felipe B. Alves[1], Heber E. Andrada[1], Bruno A. Fico[1], Júlia S. Reinaldi[1], Denise C. Tavares[1], Iara S. Squarisi[1], Gabriel Sgarbiero Montanha[2,3], Laura G. Nuevo[2], Hudson W. P. de Carvalho[2,4], Carlos A. Pérez[5], Eduardo F. Molina[1]*

[1] Universidade de Franca, Av. Dr. Armando Salles Oliveira 201, Franca, SP, 14404-600, Brazil

[2] Grupo de Estudo em Fertilizantes Especiais e Nutrição, Centro de Energia Nuclear na Agricultura, Universidade de São Paulo, Av.Centerário 303, Piracicaba, SP, 13400-970, Brazil

[3] Dipartimento di Biologia e Biotecnologie Charles Darwin, Sapienza Università degli Studi di Roma 'La Sapienza', Via dei Sardi 70, Roma, RM, 00185, Italy.

[4] Chair of Soil Science, Mohammed VI Polytechnic University, Lot 660, Ben Guerir 43150, Morocco.

[5] Brazilian Synchrotron Light Laboratory, Brazilian Centre for Research in Energy and Materials, Rua Giuseppe Máximo Scolfaro, 10000, 13083-1000 Campinas, Brazil

*Corresponding author e-mail: eduardo.molina@unifran.edu.br

Table S1: Physico-chemical properties of the test water for Zebrafish assays.

Characteristic	Unit	Mean
Air temperature	°C	25.0
Water temperature	°C	26.5
pH	-	7.2
Conductivity	$\mu M\ cm^{-1}$	62.5
Dissolved oxygen	$mg\ L^{-1}$	72.5

Table S2. Fe Kα and Kβ lines fitting output from the spectra obtained on the synchrotron-based XRF mapping of cryofixed cross-sections of cucumber seeds primed with either the negative (water) and positive controls (Fe solution) or the microPPO-Fe gel solutions. FWHM: full width at half maximum; $\sum X^2$: chi-square summing the 242400 XRF points recorded; X^2: average chi-square of each XRF point recorded.

Treatment	Replicate	Line	Energy (keV)	Group	Fit Area (counts)	FWHM	$\sum X^2$	X^2
water	1	Kα	6.391	KL2a	2.11E+08	0.182	36914	0.15
			6.404	KL3a	4.12E+08	0.183	29377	0.12
		Kβ	7.058	KM3b	9.49E+07	0.186	10308	0.04
	2	Kα	6.391	KL2a	2.62E+08	0.182	52754	0.22
			6.404	KL3a	5.14E+08	0.182	52507	0.22
		Kβ	7.058	KM3b	1.17E+08	0.186	13351	0.06
Fe-solution	1	Kα	6.391	KL2a	1.91E+08	0.191	10080	0.04
			6.404	KL3a	3.73E+08	0.191	10259	0.04
		Kβ	7.058	KM3b	8.16E+07	0.195	8349	0.03
	2	Kα	6.391	KL2a	2.91E+08	0.229	984519	4.06
			6.404	KL3a	5.70E+08	0.229	870770	3.59
		Kβ	7.058	KM3b	1.07E+08	0.236	547797	2.26
microPPO-Fe	1	Kα	6.391	KL2a	3.79E+08	0.185	35807	0.15
			6.404	KL3a	7.43E+08	0.185	34968	0.14
		Kβ	7.058	KM3b	1.65E+08	0.189	11869	0.05
	2	Kα	6.391	KL2a	3.01E+08	0.186	47681	0.20
			6.404	KL3a	5.89E+08	0.186	45854	0.19
		Kβ	7.058	KM3b	1.32E+08	0.189	12221	0.05

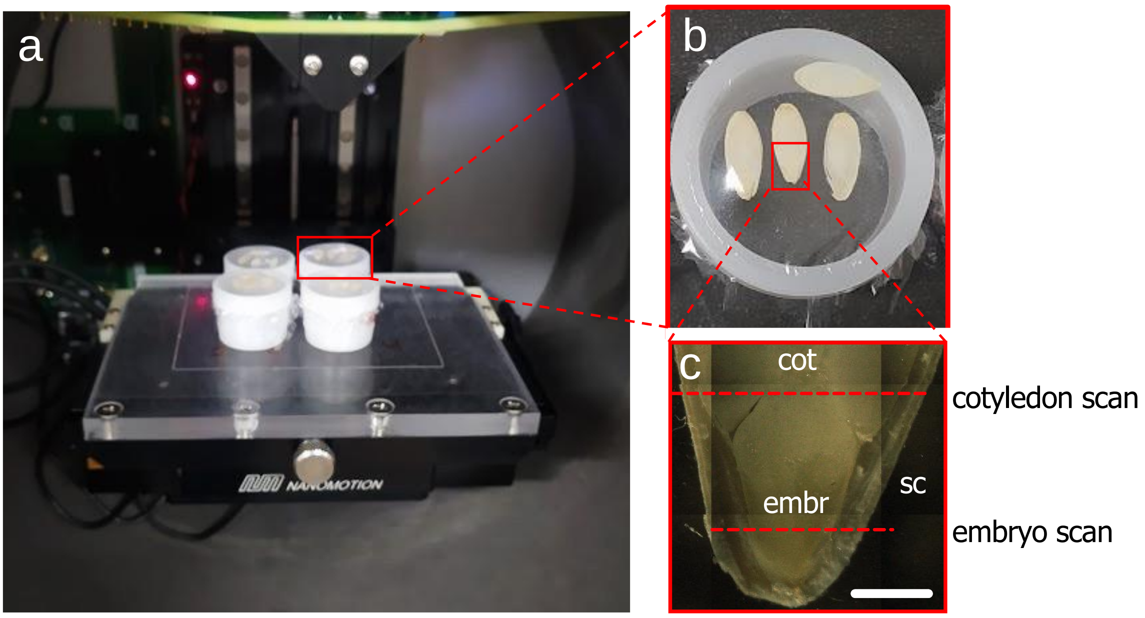

Figure S1. Details of the samples and experimental setup employed for µ-XRF assessment of Fe distribution in cucumber seeds. Scale: 1 mm.

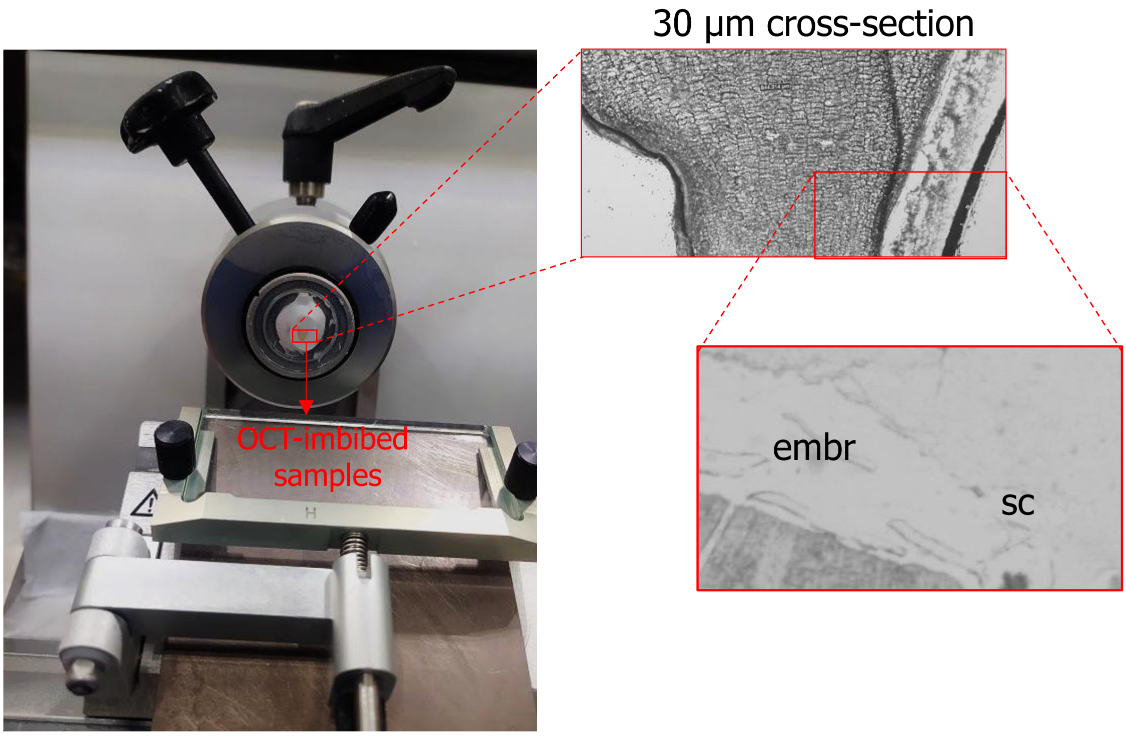

Figure S2. Details of the OCT-imbibed cryofixed cucumber seeds on the cryomicrotome, and the resulting 30-µm thick-cross-sections used for assessing the elemental distribution at the seed coat (sc) and embryo (embr) interface.

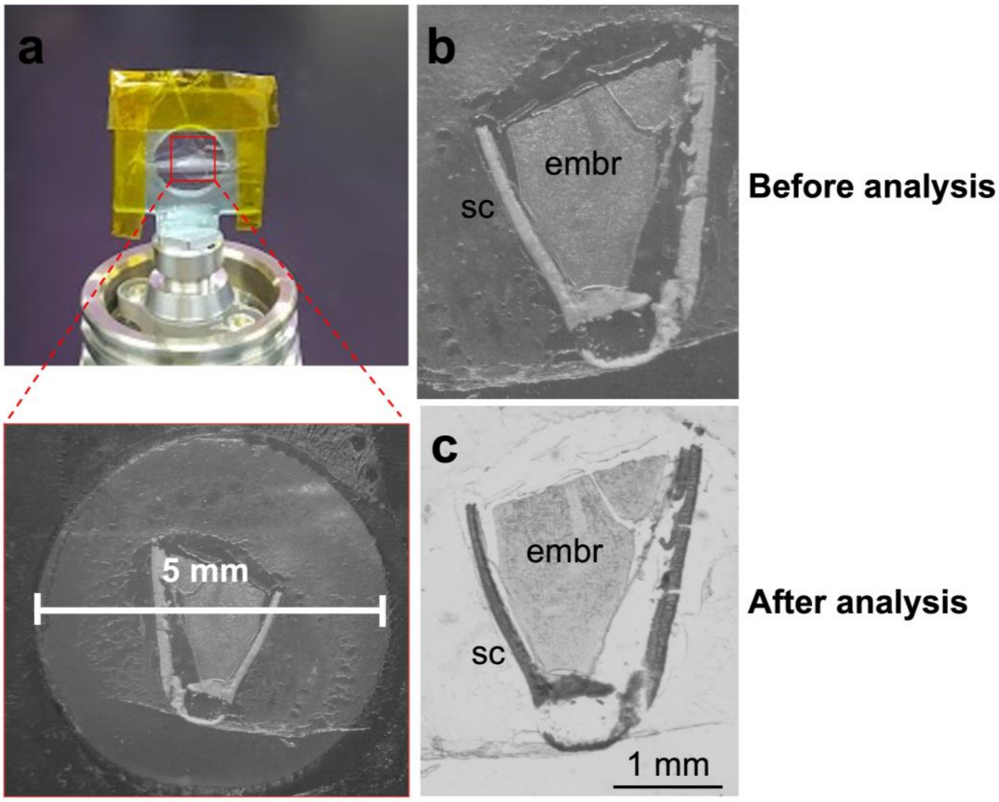

Figure S3. Details of the cryofixed cucumber seed cross-section fixed in XRF sample holder for synchrotron-based XRF measurements (a). Stereomicroscope images of a seed sample before and after the analyses (b-c), indicating its structural integrity. sc = seed coat; embr = embryo.

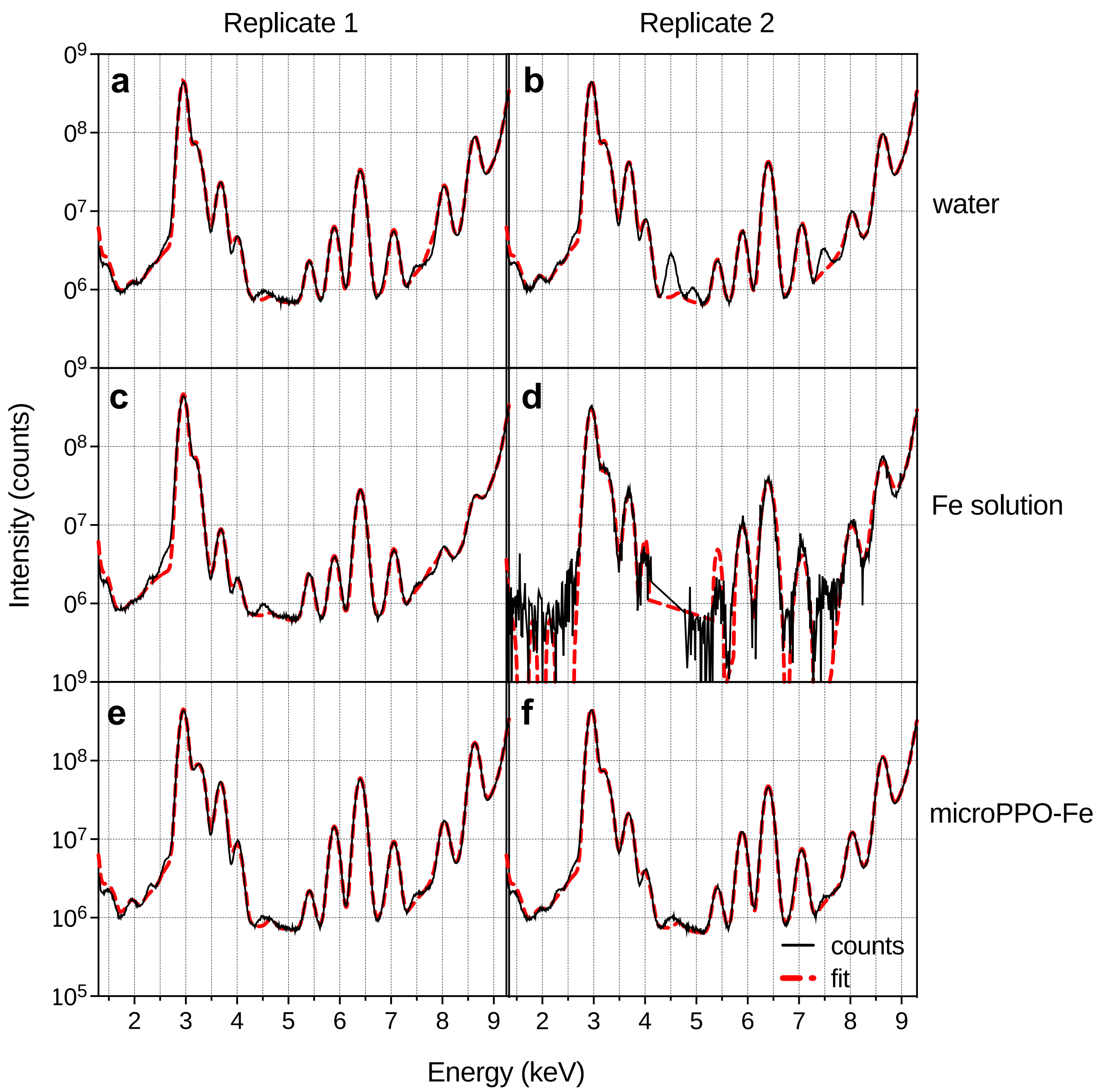

Figure S4. Spectra and fittings for the synchrotron-based XRF maps of cryofixed cross-sections of cucumber seeds primed with either the negative (water, a-b) and positive controls (Fe solution, c-d) or the microPPO-Fe gel solutions (e-f). Each spectrum represents the sum of all 242400 XRF points recorded.

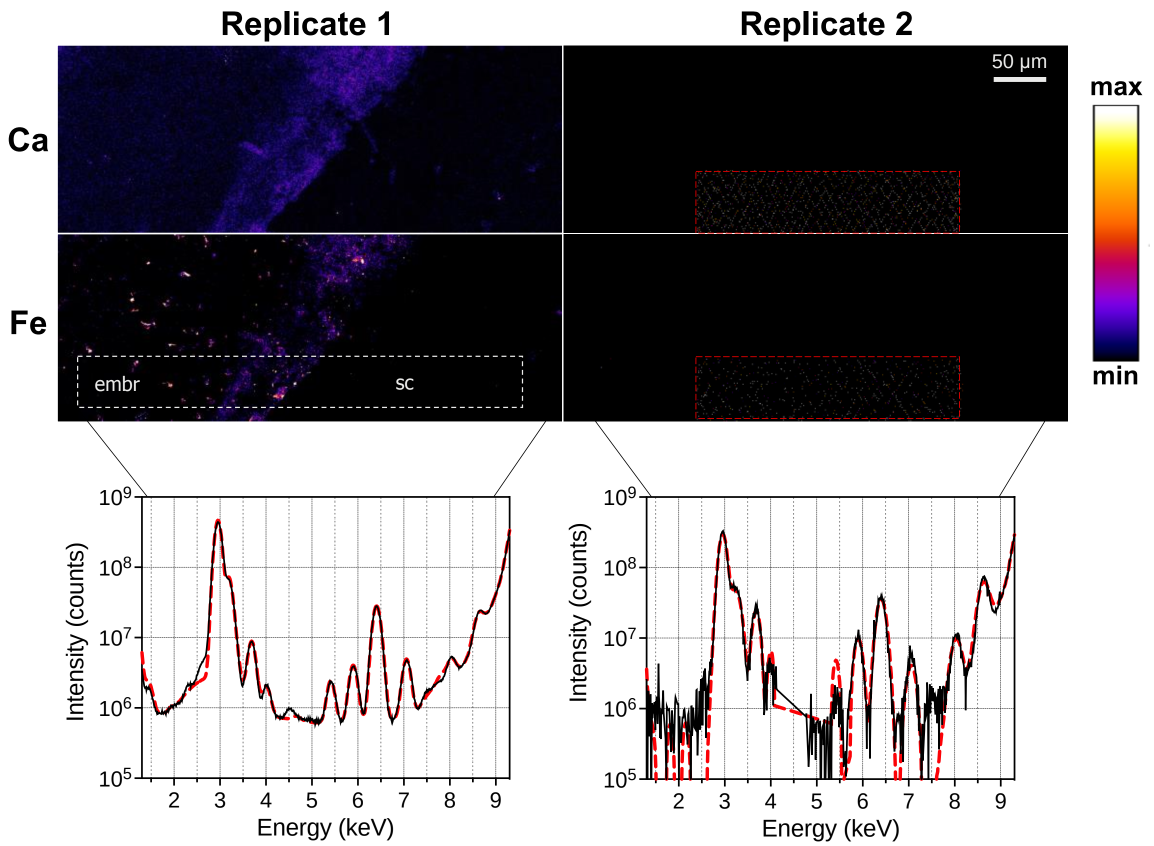

Figure S5. Synchrotron-based XRF maps and spectra of Fe distribution in cryofixed cross-sections of cucumber seeds primed with the positive control (Fe-solution). The map of replicate 2, as indicated by the red dashed box, was not properly recorded due to beamline instrumental problems. Therefore, it cannot be used for comparative analyses.

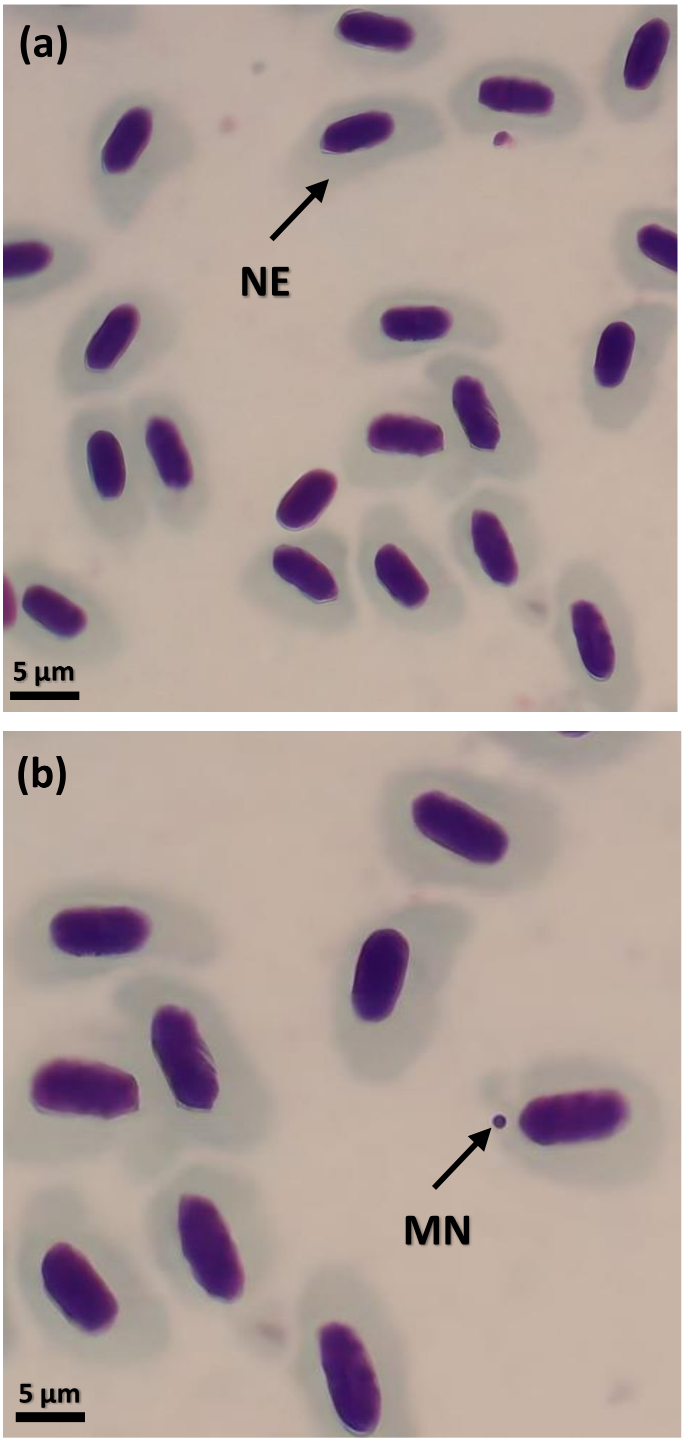

Figure S6. Blood smears of adult zebrafish (a) normal erythrocytes and (b) erythrocytic nuclear abnormalities. NE: normal erythrocyte and MN: erythrocyte with a micronucleus.

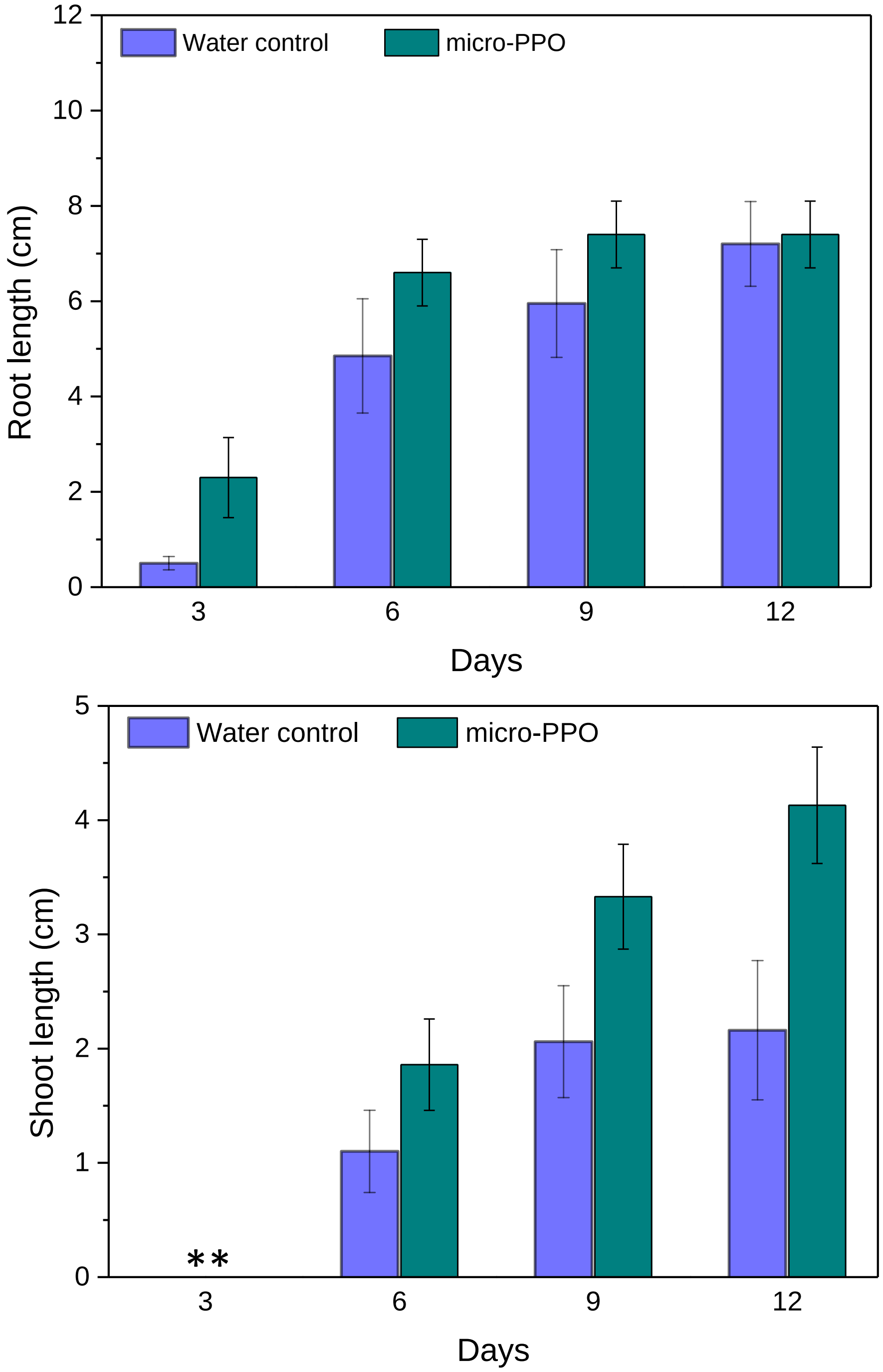

Figure S7. Initial seedling growth during 12 days after sowing in water (control) and micro-PPO gels. Evolution of the root and shoot length. ** Initial germination process with radicals emerging from the seeds. All the results represent the average of 7 Petri plates, containing 3 seeds per plate.

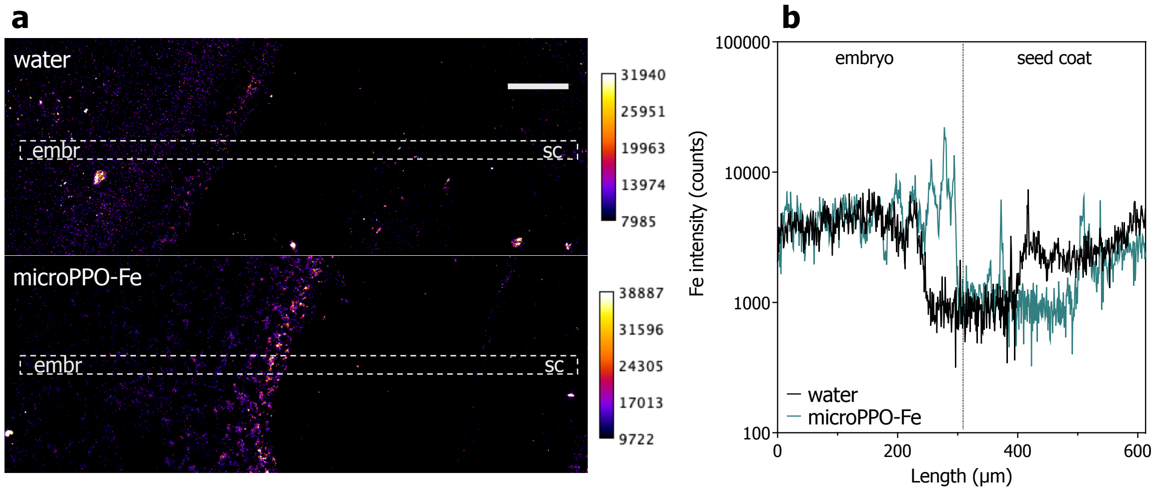

Figure S8. Biological replicate of synchrotron-based XRF maps of Fe distribution in cryofixed cross-sections of cucumber seeds primed with either the negative (water) control or the microPPO-Fe gel solutions (a). Fe profile across the seed coat (sc) and embryo (embr) interface within the dashed box (b). Scale: 50 µm.
